# Natural Killer Cells Induce CD8^+^ T Cell Dysfunction *via* Galectin-9/TIM-3 in Chronic Hepatitis B Virus Infection

**DOI:** 10.3389/fimmu.2022.884290

**Published:** 2022-06-28

**Authors:** Siyu Liu, Chang Xu, Fan Yang, Lu Zong, Yizu Qin, Yufeng Gao, Qian Su, Tuantuan Li, Ye Li, Yuanhong Xu, Meijuan Zheng

**Affiliations:** ^1^ Department of Clinical Laboratory, First Affiliated Hospital of Anhui Medical University, Hefei, China; ^2^ Anhui Center for Disease Control and Prevention, Hefei, China; ^3^ Department of Infectious Diseases, The First Affiliated Hospital of Anhui Medical University, Hefei, China; ^4^ Department of Clinical Laboratory, Second People’s Hospital of Fuyang City, Fuyang, China; ^5^ The First Affiliated Hospital of University of Science and Technology of China (USTC), Division of Life Sciences and Medicine, University of Science and Technology of China, Hefei, China

**Keywords:** CHB, NK cell, CD8^+^T cells, Gal-9, TIM-3

## Abstract

The antiviral response of natural killer (NK) cells and CD8^+^ T cells is weak in patients with chronic hepatitis B (CHB) infection. However, the specific characteristics of these cells and the association between NK cells and CD8^+^ T cell dysfunction is not well known. In this study, higher galectin-9 (Gal-9) expression was observed in circulating NK cells from CHB patients than from healthy controls and was found to contribute to NK cell dysfunction. In addition, circulating CD8^+^ T cells showed obvious dysfunction and overexpressed TIM-3, the natural receptor of Gal-9, during active CHB infection. Gal-9^+^ and Gal-9^-^ NK cells from active CHB patients were sorted and cocultured with autologous CD8^+^ T cells. The proportion of tetramer^+^CD8^+^ T cells and the cytokines production of CD8^+^ T cells were lower after cocultivation with Gal-9^+^ than with Gal-9^-^ NK cells. We showed that *in vitro* depletion of NK cells increased circulating hepatitis B virus (HBV)-specific CD8^+^ T cell responses in patients with active CHB infection. Because Gal-9 is increased in the serum of CHB patients, CD8^+^ T cells were sorted and cultured with exogenous Gal-9, resulting in lower IFN-γ, TNF-α, CD107a, and granzyme B levels, decreased expression of the activation receptor CD69, increased expression of TIM-3, and a high percentage of early apoptotic CD8^+^ T cells. Blocking Gal-9 or TIM-3 *in vitro* in a culture of peripheral blood mononuclear cells (PBMCs) stimulated with HBV peptide from active CHB patients restored CD8^+^ T cell function. However, blocking Gal-9 *in vitro* after removal of NK cells from PBMCs did not rescue CD8^+^ T cells exhaustion. Furthermore, NK and CD8^+^ T cells from active CHB patients were sorted and cocultured *in vitro*, and the exhaustion of CD8^+^ T cells were alleviated after blocking Gal-9 or TIM-3. In summary, overexpression of Gal-9 on NK cells, which interacts with TIM-3^+^CD8^+^ T cells and likely contributes to antiviral CD8^+^ T cell dysfunction, may be a potential target for the treatment of CHB patients.

## 1 Introduction

Hepatitis B virus (HBV) is a hepatotropic virus that can cause persistent infection (1). Approximately 2 billion people are infected with HBV, of whom over 350 million are chronic carriers (2). Without proper management, up to 20% of patients with chronic hepatitis B (CHB) die from HBV-induced liver diseases such as cirrhosis, hepatocellular carcinoma, and liver failure (3). Viral persistence due to a low antiviral immune response is thought to contribute to HBV infection-related pathology (4). However, the exact mechanism of HBV-mediated immunosuppression during chronic infections is not fully clarified.

Natural killer (NK) cells are important innate immune lymphocytes that serve as the first line of defense against viral infection (5). We have postulated that an increase in inhibitory receptor expression on NK cells contributes to CD8^+^ T cell dysfunction during chronic viral infection and hepatocellular carcinoma (6). During chronic HBV infection, viral persistence results in NK cell dysfunction and impaired production of cytokines such as IFN-γ (7). Our previous study (8) demonstrated that Hepatitis B e antigen (HBeAg) induces NKG2A^+^ NK cell dysfunction through the production of regulatory T cell-derived Interleukin 10 (IL-10) during CHB. The outcome of hepatic diseases is primarily dependent on antigen-specific T cell immune responses (6). Furthermore, CD8^+^ T cell plays a key role in viral elimination during HBV infection (9). When infection persists, virus-specific CD8^+^ T cells enter a state known as T cell exhaustion (10). Exhausted CD8^+^ T cells have distinct features, including overexpression of inhibitory receptors and dysfunctional cytokine signaling (11–13). In CHB patients, effector CD8^+^ T cells are in a state of multi-level exhaustion with significantly lower rates of proliferation and IFN-γ, IL-2, TNF-α, granzyme, and perforin production (14, 15). Moreover, several human and mouse studies have shown that NK cells regulate and restrict T cell immunity (16). Based on the type of immune stress, NK cells can limit T cell function by impacting perforin (17), NKp46 (18), or NKG2D (19) expression and cytokine production (20–22). Removal of NK cells *in vitro* was shown to increase HBV-specific CD8^+^ T cell responses (23). However, the mechanism underlying T cell exhaustion and the relationship between NK cell exhaustion and T cell exhaustion in CHB patients requires further study.

Galectin-9 (Gal-9) is a member of the galectin family of animal lectins with conserved carbohydrate recognition domains (CRDs) for β-galactosides. It consists of two CRDs linked by a single sequence, which cross-links glycoproteins to form a polyvalent galactose protein grid that regulates various cellular activities (24, 25). Gal-9 is widely expressed in a variety of immune and non-immune cells and exists in the membranes, cytoplasm, and nuclei (25, 26). The expression of Gal-9 on NK cells has been reported, and studies show that Gal-9^+^ NK cells are associated with decreased granzyme B, perforin, and granulysin production (27–29). During chronic viral infection, TIM-3 is continuously expressed on exhausted T cells (30). As a natural ligand for TIM-3, Gal-9 plays a key immunomodulatory role by inducing apoptosis or inhibiting effector function through interaction with TIM-3. CD8^+^ T cells overexpress TIM-3 during HIV infection, and binding of Gal-9 to TIM-3 contributes to CD8^+^ T cell failure (31, 32). Blocking TIM-3 with Gal-9 *in vitro* can rescue HIV, HCV, and HBV-specific CD8^+^ T cell exhaustion (33, 34). However, it remains unclear whether NK cells regulate CD8^+^ T cells through Gal-9/TIM-3 in CHB patients.

In this study, we found an increase in Gal-9^+^ NK cells in the peripheral blood of CHB patients compared to healthy controls, which contributed to anti-HBV CD8^+^ T cell dysfunction through Gal-9/TIM-3 axis. Overall, this study clarifies a mechanism by which NK cells induce CD8^+^ T cell exhaustion and suggests a potential treatment target for CHB infection.

## 2 Materials and Methods

### 2.1 Human Subjects

The study included 328 subjects divided into the following four groups: Tolerant CHB (n=25), Active CHB (n=117), Inactive CHB (n=40), and age- and sex-matched healthy controls (n=146; HCs). All patients were diagnosed with HBV infection and untreated while HCs were ruled out for infection with hepatitis or other diseases that could influence the study. Characteristics of the enrolled subjects are summarized in **Table 1**. Tolerant CHB patients were characterized as HBeAg-positive and had normal alanine transferase (ALT) and elevated levels of HBV DNA that were >20,0000 IU/mL; Active CHB patients were characterized as having serum ALT levels >61 U/L and HBV-DNA levels >2000 IU/mL; Inactive CHB patients were characterized by the absence of HBeAg and the presence of anti-HBe antibodies, normal ALT levels, and HBV DNA <2000 IU/mL (35). Twenty CHB patients who were receiving antiviral therapy for more than 6 months were also recruited into the study. The study was approved by the local ethics committee of The First Affiliated Hospital of Anhui Medical University.

**Table 1:** Characteristics of human subjects.

	All CHB patients	Tolerant	Active	Inactive	HC
N	182	25	117	40	146
Age (years)	39.4 ± 0.9	34.9 ± 2.1	39.7 ± 1.2	41.3 ± 1.5	35.7 ± 0.9
Male/female	108/74	8/17	79/38	21/19	69/77
ALT (U/L)	296.4 ± 34.5	28.7 ± 2.7	445.9 ± 48.3	23.6 ± 1.6	20.0 ± 0.7
HBsAg (+/-)	182/0	25/0	117/0	40/0	NA
HBeAg (+/-)	96/86	25/0	70/47	0/40	NA
HBV DNA (IU/mL), median	6.05×10^6^	2.07×10^8^	1.4×10^7^	4.01×10^2^	NA

Clinical chemical data are given as mean± SEM. HC, healthy control; NA, not applicable.

### 2.2 Serological Experiments

HBV markers, including HBsAg, HBsAb, HBeAg, HBeAb, and HBcAb, were quantified using commercial enzyme immunoassay kits (Zhongshan Bio-Tech, China). HBV DNA levels were determined by real-time PCR (Roche LightCycler480, Switzerland). Serum ALT was detected using an automatic biochemical analyzer (Cobas 8000, Roche Diagnostics GmbH, Switzerland).

### 2.3 Flow Cytometry Analysis

Peripheral blood mononuclear cells (PBMCs) were isolated using Ficoll-Isopaque (TBD) gradient centrifugation from freshly peripheral blood. Peripheral lymphocytes and CD8^+^ T cells from the *in vitro* cultures were stained with fluorochrome-conjugated antibodies, collected using a BD FACSCanto Plus flow cytometer, and analyzed with FlowJo VX (Tree Star, Ashland). The following mouse anti-human monoclonal antibodies were used: BV510-CD3, BV605-CD3, FITC-CD56, PE-CD56, BV421-CD56, APC-H7-CD8, APC-CY7-CD8, PE-CY7-CD8, BV421-TIM-3, PE-PD-1, FITC-CD158a, 647-NKP30, APC-NKP46, APC-NKG2D, FITC-CD226, PE-CY7-CD69, FITC-IFN-γ, PE- IFN-γ, PerCP-IFN-γ, FITC-TNF-α, PE-TNF-α, APC-TNF-α, PE-CY7-CD107a, BV510-CD107a, PE-Granzyme B, V450-Granzyme B, BV510-Granzyme B, 647-Perforin (BD Biosciences); FITC-NKG2A, PE-CD158b (Miltenyi Biotec); PE-CY7-NKG2A (BECKMAN); 660-Galectin-9, PE-HLA-E (eBioscience); PE-TIM-3, PE-KIR3DL1 (R&D); APC-TIGIT, BV605-TIGIT (BioLegend), FITC-CD69 (Thermo fisher).

For intracellular cytokines staining, PBMCs were incubated with DMEM (HyClone) containing 5% FBS (Gibco) with 50 ng/mL phorbol 12-myristate 13-acetate, 1 µg/mL ionomycin, and 2.5 µg/mL monensin (all from Sigma-Aldrich, USA) at 37°C in a 5% CO_2_ incubator for 4 h. CD107a antibody was added at the start of the incubation. Cells were then stained with the membrane markers. Afterwards, the cells were fixed, permeabilized, and stained with antibodies against IFN-γ, TNF-α, granzyme B, and perforin.

To detect CD8^+^ T cells apoptosis after *in vitro* culture, cells were stained with BV510-CD3 and PE-CY7/APC-H7-CD8 for 30 min. After washing with 1× Annexin V binding buffer (BioLegend), the cells were suspended in 100 μL Annexin V binding buffer and stained with FITC-Annexin V (BD Biosciences) for 15 min. The staining was terminated using 100 μL PBS, and 7-AAD (BioLegend) was added to exclude dead cells before analysis using a BD FACSCanto Plus flow cytometer.

To detect HBV-specific CD8^+^ T cells, PBMCs cultured *in vitro* were harvested and stained with PE-tetramer (core18-27) for 1 h. After washing with PBS, the cells were incubated with BV510-CD3, PE-CY7-CD8 for 30 min, and 7-AAD (BioLegend) was added prior to analysis.

### 2.4 Cell Sorting

NK cells were purified from PBMCs using a human NK cell Isolation Kit (Miltenyi Biotec, 130-092-957) according to the manufacturer’s instructions. The purity of the isolated NK cells was > 90%, and the PBMCs-ΔNK were the cells left after depletion of NK cells.

PBMCs isolated from the fresh blood of active CHB patients were stained with BV510-CD3, BV421-CD56, APC-H7-CD8, and 660-Gal-9 and NK, Gal-9^+/-^ NK, CD8^+^ T cells were purified using a FACS Aria II (BD Biosciences). The purity of isolated cells was >95%.

### 2.5 Cell Culture

#### 2.5.1 Co-Culture of CD8^+^T Cells With Gal-9^+^ or Gal-9^-^NK cells

CD8^+^ T cells (5 × 10^4^) and Gal-9^+^ NK cells (5 × 10^4^) or Gal-9^-^ NK cells (5 × 10^4^) at a ratio of 1:1 were co-cultured in DMEM with 10% FBS (containing 200 U/mL penicillin, 0.2 mg/mL streptomycin, and 100 mM Hepes buffer) and IL-2 (100 IU/mL) with 5 μg/mL anti-CD3 and 5 μg/mL anti-CD28 at 37°C in a 5% CO_2_ incubator for 3 days. The percentage of HBV-specific CD8^+^ T cells and the function of CD8^+^ T cells were assessed.

#### 2.5.2 Co-Culture of CD8^+^T Cells With Exogenous Gal-9

CD8^+^ T cells (1× 10^5^) purified by FACS were cultured in DMEM with 10% FBS (containing 200U/mL penicillin, 0.2 mg/mL streptomycin, and 100 mM Hepes buffer) and IL-2 (100IU/mL) with 5 µg/mL anti-CD3, 5 µg/mL anti-CD28 and 5 ng/mL exogenous recombinant human Gal-9 (rhGal-9) (R&D Systems) at 37°C in a 5% CO2 incubator for 3 days. Monensin and CD107a monoclonal antibody were added during the last 4 h. TIM-3, CD69, IFN-γ, TNF-α, CD107a, and granzyme B expression along with CD8^+^ T cell apoptosis were assessed.

#### 2.5.3 PBMCs Culture

PBMCs (2 × 10^6^) and PBMCs with depletion of NK cells (2 × 10^6^) were resuspended in complete medium (DMEM containing 10% FBS, 200U/mL penicillin, 0.2 mg/mL streptomycin, and 100 mM Hepes buffer) and stimulated with individual HBV peptides (core18-27, FLPSDFFPSV) (2 µg/mL) and recombinant interleukin-2 (IL-2) (100 IU/mL). Cells were cultured for 10 days and fed twice weekly with complete medium and IL-2. On day 10, monensin and CD107a monoclonal antibody were added, and IFN-γ, TNF-α, CD107a, granzyme B, and perforin expressions by CD8^+^ T cells were detected.

#### 2.5.4 Blocking Assays

PBMCs (2 × 10^6^) were resuspended in complete medium (DMEM containing 10% FBS, 200 U/mL penicillin, 0.2 mg/mL streptomycin, and 100 mM Hepes buffer) and stimulated with individual HBV peptides (core18-27, FLPSDFFPSV) (2 µg/mL) and recombinant interleukin-2 (IL-2) (100 IU/mL) in the presence of 10 µg/mL Gal-9 inhibitor (9M1-3, BioLegend), TIM-3 inhibitor (10 µg/mL; F38-2E2, BioLegend), and IgG (BioLegend). Cells were cultured for 10 days and fed twice weekly with complete medium and IL-2. On day 10, monensin and CD107a monoclonal antibody were added, and IFN-γ, TNF-α, CD107a, granzyme B, and perforin expressions by CD8^+^ T cells were detected.

PBMCs with depletion of NK cells (2 × 10^6^) were resuspended in complete medium (DMEM containing 10% FBS, 200 U/mL penicillin, 0.2 mg/mL streptomycin, and 100 mM Hepes buffer) and stimulated with individual HBV peptides (core18-27, FLPSDFFPSV) (2 µg/mL) and recombinant interleukin-2 (IL-2) (100IU/mL) in the presence of 10 µg/mL Gal-9 inhibitor (9M1-3, BioLegend) or IgG (BioLegend). Cells were cultured for 10 days and fed twice weekly with complete medium and IL-2. On day 10, monensin and CD107a monoclonal antibody were added, and IFN-γ, TNF-α, CD107a, granzyme B, and perforin expressions by CD8^+^ T cells were detected.

CD8^+^ T cells (1 × 10^5^) purified by FACS were cultured in DMEM with 10% FBS (containing 200 U/mL penicillin, 0.2 mg/mL streptomycin, and 100 mM Hepes buffer) and IL-2 (100 IU/mL) in the presence of 5 µg/mL anti-CD3, 5 µg/mL anti-CD28, and 10 µg/mL Gal-9 inhibitor (9M1-3, BioLegend) or IgG (BioLegend) at 37°C in a 5% CO_2_ incubator for 3 days. Monensin and CD107a monoclonal antibody were added during the last 4 h, and IFN-γ, TNF-α, CD107a, granzyme B, and perforin were analyzed.

CD8^+^ T cells (5 × 10^4^) and NK cells (5 × 10^4^) at a ratio of 1:1 were cocultured in DMEM with 10% FBS (containing 200 U/mL penicillin, 0.2 mg/mL streptomycin, and 100 mM Hepes buffer) and IL-2 (100 IU/mL) in the presence of 5 µg/mL anti-CD3 and 5 µg/mL anti-CD28 at 37°C in a 5% CO_2_ incubator for 3 days. Gal-9 inhibitor (10 µg/mL; 9M1-3, BioLegend), TIM-3 inhibitor (10 µg/mL; F38-2E2, BioLegend), and a corresponding IgG control (BioLegend) were added to the culture. Monensin and CD107a monoclonal antibody were added during the last 4 h, and the expressions of IFN-γ, TNF-α, CD107a, granzyme B, and perforin and CD8^+^ T cell apoptosis were assessed.

### 2.6 Quantitative Real-Time PCR (qRT-PCR)

Total RNA was extracted from NK cells using the RNeasy Mini Kit (QIAGEN) in CHB patients and HCs and converted to cDNA using the RevertAid First Strand cDNA Synthesis Kit (Thermo Fisher Scientific). The forward and reverse primers were as follows: 5′-TGCAACACGAGGCAGAACG-3′ and 5′-CACAGAGCCATTGACGGAGAT-3′ for galectin-9, 5′-GAGTCAACGGATTTGGTCGT-3′ and 5′-TTGATTTTGGAGGGATCTCG-3′ for GAPDH. Gene expression was measured using a real-time PCR machine (cobas z 480, Switzerland) and PowerUp SYBR Green Master Mix (Thermo Fisher Scientific). The mRNA level of Galectin-9 was normalized to that of GAPDH, and the relative expression of mRNA was calculated using the 2^−ΔΔCt^ method.

### 2.7 ELISA

The level of serum galectin-9 was measured using a Human Galectin-9 Quantikine ELISA Kit (R&D). In brief, reagents, standard dilutions, and samples were prepared according to the manufacturer’s instructions. Assay diluent (100 µL) was added to each well followed by 100 µL of a prepared standard, control, or 4 times diluted sample, and the plate was incubated for 2 h on a microplate shaker. After washing, 200 µL of conjugate were added to each well, and the plate was incubated for 2 h on the shaker. After additional washing, 200 µL substrate solution was added to each well, the plate was incubated for 30 min in the dark, 50 µL stop solution was added, and the plate was read at 450 nm within 30 min. All procedures were performed at room temperature.

### 2.8 Statistics Analysis

The data were analyzed using Graphpad Prism 6.0 and shown as the mean ± SEM. A Student’s t-test was used for two-group comparisons and a one-way ANOVA was used for three-group and four-group comparisons. p <0.05 was considered significant.

## 3 Results

### 3.1 Expression of Gal-9 Is Increased and Associated With Functional Impairment in NK Cells of CHB Patients

**Figure 1:**
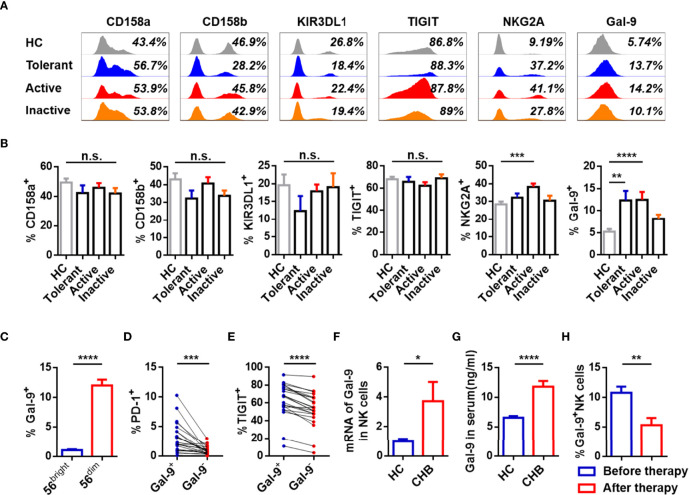
Expression of Gal-9 is increased and associated with functional impairment in NK cells of CHB patients. **(A)** Representative flow cytometry plots showing expression of the inhibitory receptors, CD158a, CD158b, KIR3DL1, TIGIT, NKG2A, and Gal-9 on circulating NK cells in the Tolerant, Active, and Inactive groups of CHB patients and HCs. **(B)** Comparison of the proportion of receptors expressed on circulating NK cells in **(A)**. **(C)** Comparison of Gal-9 expression in circulating CD56^bright^ NK cells and CD56^dim^ NK cells in CHB patients. **(D)** Comparison of the PD-1 expression levels in circulating Gal-9^+^ NK cells and Gal-9^-^ NK cells in patients with CHB. **(E)** Comparison of the TIGIT levels in circulating Gal-9^+^ NK cells and Gal-9^-^ NK cells in patients with CHB. **(F)** Gal-9 mRNA expression in circulating NK cells from CHB patients and HCs. **(G)** Gal-9 expression in serum from CHB patients and HCs. **(H)** Comparison of the Gal-9 expression levels in NK cells in peripheral blood of CHB patients with or without antiviral treatment, respectively. Results are expressed as the mean ± SEM, and the number of samples (n) in each group was ≥ 3. One-way ANOVA test was conducted for four-group comparisons. Groups with significant differences are marked, and the unmarked paired groups have no differences. Unpaired t-test was used to compare two independent groups. A paired t-test was used to compare paired samples. *P < 0.05; **P < 0.01; ***P < 0.001; ****P < 0.0001; n.s., not significant.

To study the phenotype of NK cells, expression of inhibitory receptors (CD158a, CD158b, KIR3DL1, TIGIT, NKG2A, Gal-9) and activating receptors (NKP30, NKP46, NKG2D, CD226) was measured on circulating NK cells from patients with different stages of CHB and healthy controls (HCs). Gating strategies of lymphocytes and NK cells were showed in **Supplementary Figure 1A**. Active CHB patients had a higher number of NKG2A^+^ NK cells than HCs, and patients at all stages, especially tolerant and active periods of CHB had elevated Gal-9^+^ NK cells than HCs. A representative FACS plot of Gal-9^+^ and Gal-9^-^ NK cells gating is shown in **Supplementary Figure 1B**. The expression of other receptors on NK cells, including CD158a, CD158b, KIR3DL1, TIGIT, NKP30, NKP46, NKG2D, and CD226, was equivalent between the groups (**Figures 1A, B** **;** **Supplementary Figures 2A, B**). Furthermore, Gal-9 was more highly expressed on CD56^dim^ than CD56^bright^ NK cells from CHB patients (**Figure 1C**).

To assess the phenotypic characteristics of Gal-9^+^ NK cells, PD-1 and TIGIT expression on Gal-9^+/-^ NK cells were analyzed in CHB patients. The gating strategies to separate Gal-9^+^ NK and Gal-9^-^ NK cells from PBMCs were detailed **Supplementary Figure 1C**. Gal-9^+^ NK cells from CHB patients expressed higher levels of PD-1 and TIGIT than Gal-9^-^ NK cells (**Figures 1D, E**).

We confirmed the expression Gal-9 on NK cells from CHB patients by measuring Gal-9 mRNA in total NK cells and found that Gal-9 mRNA was higher in NK cells from CHB patients than HCs (**Figure 1F**). Moreover, Gal-9 levels were significantly higher in the serum of CHB patients than HCs (**Figure 1G**). In addition, the 15 CHB patients who had received antiviral treatment for more than 6 months had significantly lower Gal-9 expression than CHB patients without treatment (**Figure 1H**); NKG2A^+^ NK cells were also reduced in patients after antiviral treatment, as described previously (8).

**Figure 2:**
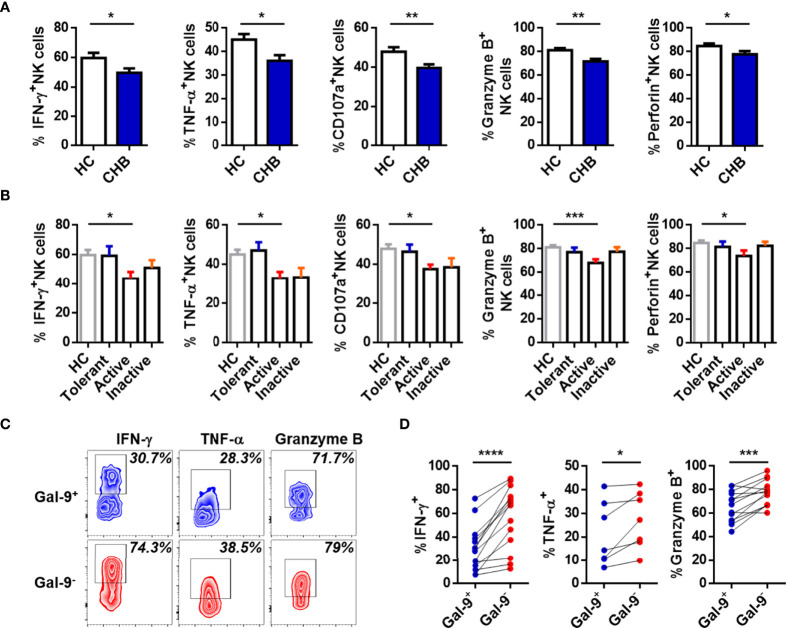
Gal-9^+^ NK cells are impaired relative to Gal-9^-^ NK cells in CHB patients. **(A)** Comparison of the expression of IFN-γ, TNF-α, CD107a, granzyme B, and perforin in circulating NK cells from CHB patients and HCs. **(B)** Comparison of the expression of IFN-γ, TNF-α, CD107a, granzyme B, and perforin in circulating NK cells from Tolerant, Active, and Inactive CHB patients and HCs. **(C)** Representative flow cytometry plots showing the expression of IFN-γ, TNF-α, and granzyme B in circulating Gal-9^+^ NK cells and Gal-9^-^ NK cells from CHB patients. **(D)** Comparison of the percentages of IFN-γ, TNF-α, and granzyme B secreted by Gal-9^+^ NK cells and Gal-9^-^ NK cells in peripheral blood from CHB patients. Results are expressed as the mean ± SEM, and the number of samples (n) in each group was ≥ 3. One-way ANOVA test was conducted for four-group comparisons. Groups with significant differences are marked, and the unmarked paired groups have no differences. An unpaired t-test was used to compare two independent groups. A paired t-test was used to compare paired samples. *P < 0.05; **P < 0.01; ***P < 0.001; ****P < 0.0001.

The function of NK cells in CHB patients was also assessed. Expression of IFN-γ, TNF-α, CD107a, granzyme B, and perforin were lower in circulating NK cells from total CHB patients than HCs (**Figure 2A**). Interestingly, the only difference between any two study groups was between active CHB patients and HCs (**Figure 2B**). These data suggest that compared with HCs, circulating NK cells were most dysfunctional in active CHB patients. Furthermore, the function of Gal-9^+^ and Gal-9^-^ NK cells was also determined. IFN-γ, TNF-α, and granzyme B production was significantly lower in Gal-9^+^ than Gal-9^-^ NK cells (**Figures 2C, D**). There was no difference in the expression of CD107a and perforin between Gal-9^+^ and Gal-9^-^ NK cells (data not shown). These results demonstrate that Gal-9 expression is increased and associated with functional impairment of NK cells from CHB patients.

### 3.2 CD8^+^ T Cells Are Functionally Exhausted During the Active Phase of CHB Infection

**Figure 3:**
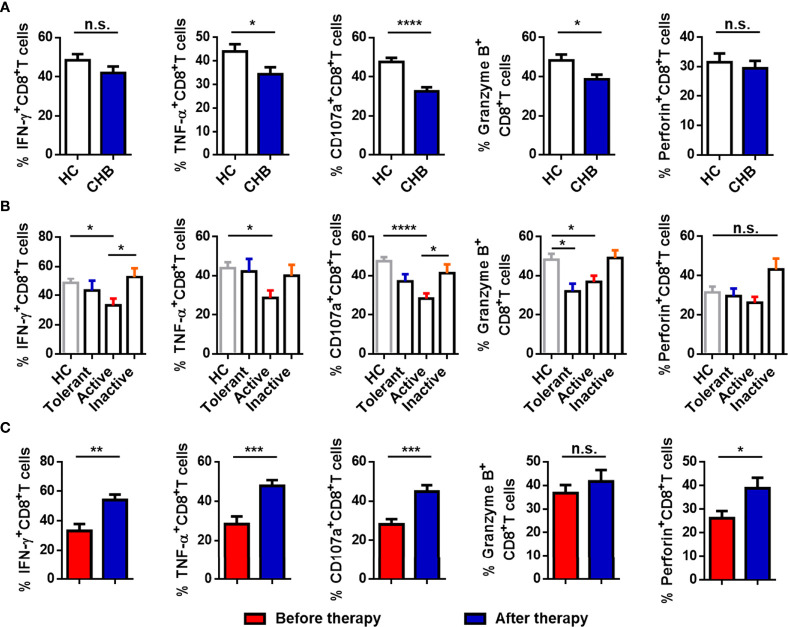
CD8^+^ T cells are functionally exhausted in active CHB patients. **(A)** Comparison of the expression of IFN-γ, TNF-α, CD107a, granzyme B, and perforin in circulating CD8^+^ T cells from CHB patients and HCs. **(B)** Comparison of the expression of IFN-γ, TNF-α, CD107a, granzyme B, and perforin in circulating CD8^+^ T cells from Tolerant, Active, and Inactive CHB patients and HCs. **(C)** Comparison of the percentages of functional molecules, including IFN-γ, TNF-α, CD107a, granzyme B, and perforin, in circulating CD8^+^ T cells from the untreated and treated CHB patients. Results are expressed as the mean ± SEM, and the number of samples (n) in each group was ≥ 3. One-way ANOVA test was conducted for four-group comparisons. Groups with significant differences are marked, and the unmarked paired groups have no differences. An unpaired t-test was used to compare two independent groups. *P < 0.05; **P < 0.01; ***P < 0.001; ****P < 0.0001; n.s., not significant.

We evaluated the function of circulating CD8^+^ T cells. As shown in **Figure 3A**, the production of TNF-α, CD107a, and granzyme B in CD8^+^ T cells from CHB patients was significantly decreased compared with HCs. The percentage of IFN-γ^+^ and perforin^+^CD8^+^ T cells was also lower, but the differences in the proportions of these cells in CHB patients and HCs were not significant. CHB patients were divided into three groups (Active, Tolerant, and Inactive), and responses were compared between the groups and with the HCs. The production of IFN-γ, TNF-α, CD107a, and granzyme B by CD8^+^ T cells was obviously lower in active CHB patients than in HCs. The percentage of granzyme B^+^CD8^+^ T cells was also decreased in tolerant CHB patients relative to HCs, and the percentage of IFN-γ^+^CD8^+^ T cells and CD107a^+^CD8^+^ T cells was lower in active than inactive CHB patients. There were no differences between the other two groups (**Figure 3B**). Moreover, cytokine production by circulating CD8^+^ T cells was markedly higher in CHB patients who had received antiviral treatment for more than 6 months than in active CHB patients without treatment (**Figure 3C**). Taken together, cytokine production by CD8^+^ T cells was impaired in CHB patients, particularly active CHB patients, compared with HCs, and the function of CD8^+^ T cells in active CHB patients was restored after antiviral treatment.

### 3.3 Gal-9/TIM-3 Axis Promotes CD8^+^ T Cell Immune Exhaustion in Active CHB Patients

**Figure 4:**
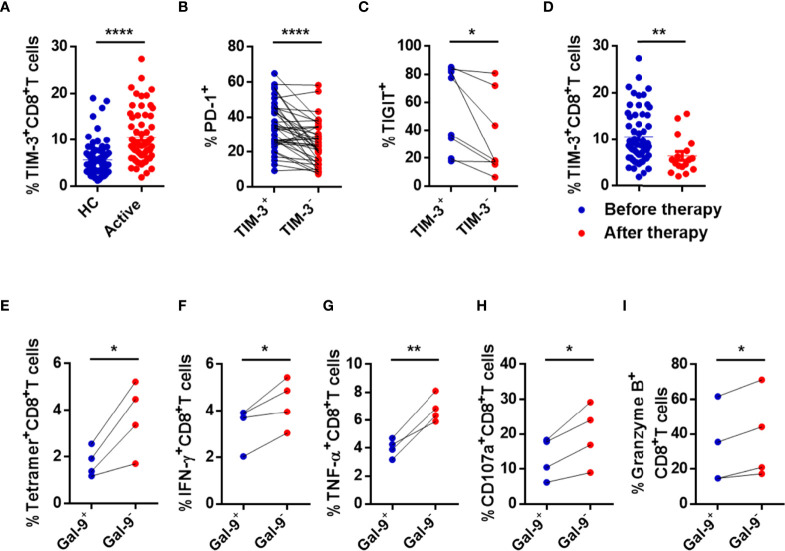
CD8^+^ T cells are immune exhausted through the Gal-9/TIM-3 axis in active CHB patients. **(A)** Comparison of the levels of TIM-3 expressed in circulating CD8^+^ T cells in active CHB patients and HCs. **(B)** The percentages of PD-1 expressed in TIM-3^+^CD8^+^ T cells and TIM-3^-^CD8^+^ T cells in peripheral blood from active CHB patients. **(C)** The percentages of TIGIT expressed on TIM-3^+^CD8^+^ T cells and TIM-3^-^CD8^+^ T cells in peripheral blood from active CHB patients. **(D)** The percentage of TIM-3 expressed on CD8^+^ T cells in peripheral blood from active CHB patients with or without antiviral treatment, respectively. **(E–I)** Gal-9^+^ NK, Gal-9^-^ NK, and CD8^+^ T cells were sorted from active CHB patients, cocultured *in vitro*, and stimulated with anti-CD3/anti-CD28 for 3 days. The frequency of tetramer^+^CD8^+^ T cells **(E)** and the production of the cytokines IFN-γ, TNF-α, CD107a, and granzyme B **(F-I)** by CD8^+^ T cells were analyzed. Results are expressed as the mean ± SEM, and the number of samples (n) in each group was ≥ 3. Unpaired t-test was used to compare two independent groups. A paired t-test was used to compare paired samples. *P < 0.05; **P < 0.01; ****P < 0.0001.

Based on previous results and the regulatory function of NK cells on CD8^+^ T cells, the relationship between impaired NK cells and exhausted CD8^+^ T cells from active CHB patients was assessed. Expression of HLA-E, a ligand of NKG2A, and TIM-3, a Gal-9 receptor, in CD8^+^ T cells from active CHB patients and HCs was measured. The gating strategies of lymphocytes and NK cells and representative flow cytometry plots of HLA-E and TIM-3 are shown in **Supplementary Figure 3A**. Of note, because our previous results (**Figures 1** **–** **3** **)** showed that NK and CD8^+^ T cells were most exhausted in active CHB patients, only cells from active patients were tested. The percentage of TIM-3^+^CD8^+^ T cells was significantly higher in active CHB patients compared with HCs (**Figure 4A**), while HLA-E expression in CD8^+^ T cells was not significant (**Supplementary Figure 3B**). Furthermore, TIM-3^+^CD8^+^ T cells had higher levels of PD-1 and TIGIT than TIM-3^-^CD8^+^ T cells in active CHB patients (**Figures 4B, C**). The detailed gating strategies to separate TIM-3^+^ and TIM3^-^CD8^+^ T cells are shown in **Supplementary Figure 3C**. In addition, the proportion of TIM-3^+^CD8^+^ T cells was lower in CHB patients who had received antiviral treatment for more than 6 months (**Figure 4D**).

To determine whether NK cells induce CD8^+^ T cell dysfunction through the Gal-9/TIM-3 pathway, we cocultured Gal-9^+^ NK cells or Gal-9^-^ NK cells with CD8^+^ T cells from active CHB patients *in vitro* and then determined the percentage of HBV-specific CD8^+^ T cells (tetramer^+^CD8^+^ T cells) and assessed the function of CD8^+^ T cells. The frequency of tetramer^+^CD8^+^ T cells and the production of IFN-γ, TNF-α, CD107a, and granzyme B by CD8^+^ T cells were significantly lower after coculture with Gal-9^+^ NK *versus* Gal-9^-^ NK cells (**Figures 4E–I**). The perforin expression was also lower after coculture with Gal-9^+^ NK cells than with Gal-9^-^ NK cells, but with no significant difference (data was not shown). The detailed gating strategies to separate Gal-9^+^ NK and Gal-9^-^ NK cells and the purity after sorting are shown in **Supplementary Figure 3D**.

**Figure 5:**
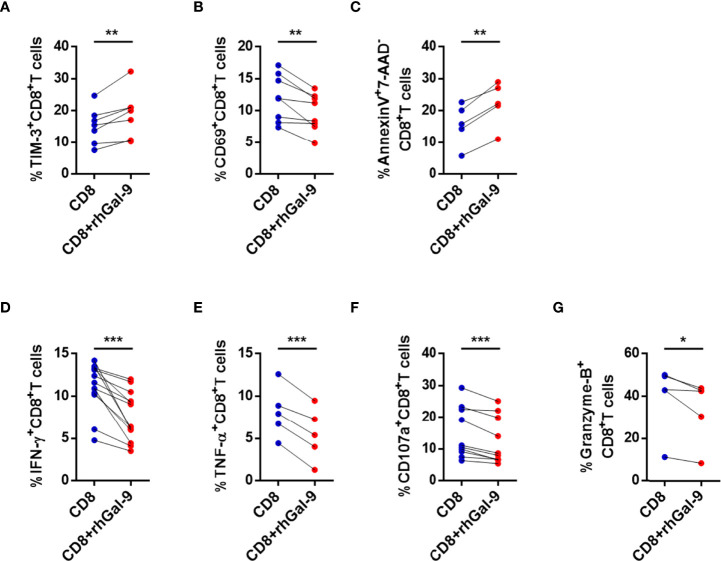
Exogenous Gal-9 induces circulating CD8^+^ T cell exhaustion in active CHB patients. **(A–G)** Coculture of CD8^+^ T cells from active CHB patients and rhGal-9 for 3 days and stimulation with anti-CD3/anti-CD28. **(A)** The percentage of TIM-3^+^CD8^+^ T cells after culture with or without rhGal-9. **(B)** The percentage of CD69^+^CD8^+^ T cells after culture with or without rhGal-9. **(C)** Comparison of CD8^+^ T cell early apoptosis after culture with and without rhGal-9. **(D–G)** Comparison of the expression of IFN-γ, TNF-α, CD107a, and granzyme B in CD8^+^ T cells from active CHB patients after culture with and without rhGal-9. Results are expressed as the mean ± SEM, and the number of samples (n) in each group was ≥ 3. A paired t-test was used to compare paired samples. *P < 0.05; **P < 0.01; ***P < 0.001.

To test whether NK cells are a source of Gal-9, exogenous Gal-9 (rhGal-9) was substituted for NK cells and cocultured with CD8^+^ T cells from active CHB patients. The expression of TIM-3 in CD8^+^ T cells was significantly increased after coculture with rhGal-9 (**Figure 5A**). In addition, CD69, IFN-γ, TNF-α, CD107a, and granzyme B expressions were significantly decreased in CD8^+^ T cells in the experimental groups compared with the controls (**Figures 5B, D–G**). The perforin production was also lower after coculture with rhGal-9, but with no significant difference (data was not shown). Furthermore, early apoptosis (Annexin V^+^7-AAD^-^) of CD8^+^ T cells increased after coculture with rhGal-9 (**Figure 5C**). In summary, CD8^+^ T cells from active CHB patients are likely to be impaired by NK cells through the Gal-9/TIM-3 but not the NKG2A/HLA-E pathway.

### 3.4 Gal-9 Expression in NK Cells Induces TIM-3^+^CD8^+^ T Cell Dysfunction

**Figure 6:**
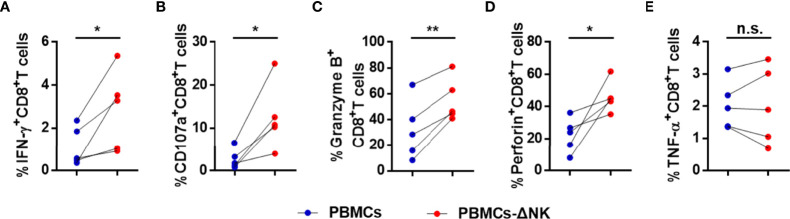
CD8^+^ T cell responses are suppressed in a NK cell-dependent manner in active CHB patients. PBMCs or PBMCs depleted of NK cells (PBMCs-ΔNK) were cultured *in vitro* and stimulated with HBV peptide (core18-27) for 10 days. The production of the cytokines IFN-γ, TNF-α, CD107a, granzyme B, and perforin **(A–E)** by CD8^+^ T cells was analyzed. Results are expressed as the mean ± SEM, and the number of samples (n) in each group was ≥ 3. A paired t-test was used to compare paired samples. *P < 0.05; **P < 0.01; n.s., not significant.

**Figure 7:**
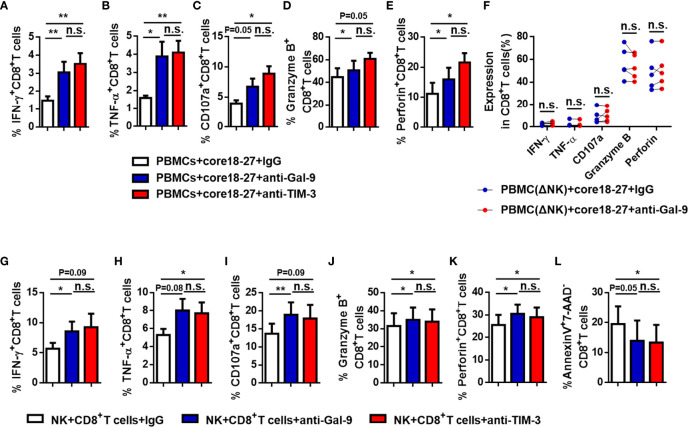
The function of anti-HBV CD8^+^ T cells in active CHB patients was restored after blocking the Gal-9/TIM-3 pathway. **(A-E)** PBMCs isolated from active CHB patients were cultured *in vitro* with anti-human Gal-9 antibody, anti-human TIM-3 antibody, or control IgG and stimulated with HBV peptide (core18-27) for 10 days. The function of HBV-specific CD8^+^ T cells was analyzed. **(F)** PBMCs depleted of NK cells (PBMCs-ΔNK) were cultured *in vitro* with anti-Gal-9 or control IgG and stimulated with HBV peptide (core18-27) for 10 days. The production of the cytokines IFN-γ, TNF-α, CD107a, granzyme B, and perforin by CD8^+^T cells was analyzed. **(G–L)** NK and CD8^+^ T cells were sorted from active CHB patients, cocultured *in vitro* with anti-human Gal-9 antibody, anti-human TIM-3 antibody, or control IgG and stimulated with anti-CD3/anti-CD28 for 3 days. The production of the cytokines IFN-γ, TNF-α, CD107a, granzyme B, and perforin by CD8^+^ T cells **(G–K)** and early CD8^+^ T cell apoptosis **(L)** were analyzed. Results are expressed as the mean ± SEM, and the number of samples (n) in each group was ≥ 3. One-way ANOVA test was conducted for three-group comparisons. A paired t-test was used to compare paired samples. *P < 0.05; **P < 0.01; n.s., not significant.

To further verify our suspicion that NK cells impair CD8^+^ T cell responses directly, we depleted NK cells from PBMCs of active CHB patient, cultured the latter *in vitro*, stimulated with HBV peptide (core18-27) for 10 days. IFN-γ, CD107a, granzyme B, and perforin expressions by CD8^+^ T cells were increased after depletion and TNF-α expression had no significant difference (**Figures 6A–E**). Then, we blocked the Gal-9/TIM-3 pathway *in vitro* and observed the impact on CD8^+^ T cells. PBMCs isolated from active CHB patients were stimulated with HBV peptide (core18-27) in the presence of anti-human Gal-9 antibody, anti-human TIM-3 antibody, or IgG for 10 days. We found that IFN-γ, TNF-α, granzyme B and perforin production by HBV-specific CD8^+^ T cells were increased after blocking Gal-9, and IFN-γ, TNF-α, CD107a and perforin production by HBV-specific CD8^+^ T cells were increased after blocking TIM-3 (**Figures 7A–E**), suggesting that TIM-3 blockade treatment had similar effect as the Gal-9 blockade treatment. However, IFN-γ, TNF-α, CD107a, granzyme B, and perforin production by CD8^+^ T cells were not altered after blocking Gal-9 while culturing the PBMCs depleted of NK cells *in vitro* (**Figure 7F**). Moreover, CD8^+^ T cells alone treated with anti-Gal-9 *in vitro* showed no functional restoration (**Supplementary Figures 4A–E**). The above results indicate that anti-Gal-9 blocking requires NK cells in the culture to exert its effect.

We further purified NK cells and CD8^+^ T cells from PBMCs of active CHB patients and cocultured them *in vitro*, stimulated with anti-CD3/anti-CD28, and blocking with anti-human Gal-9 antibody, anti-human TIM-3 antibody, or IgG for 3 days. We found that IFN-γ, CD107a, granzyme B, and perforin production increased after blocking Gal-9, and TNF-α, granzyme B, and perforin production increased after blocking TIM-3 (**Figures 7G–K**). And CD8^+^ T cell early apoptosis decreased after blocking TIM-3 (**Figure 7L**). Thus, blocking of TIM-3 has similar effect as blocking Gal-9, and NK cells negatively regulate CD8^+^T cells through the Gal-9/TIM-3 pathway.

In conclusion, these findings indicate that circulating CD8^+^ T cells from active CHB patients were exhausted. In addition, CD8^+^ T cells were impaired by NK cells through the Gal-9/TIM-3 pathway, and the function of both total and HBV-specific CD8^+^ T cells was restored after blocking the Gal-9/TIM-3 pathway *in vitro*, showing that the antiviral ability of CD8^+^ T cells was recovered.

## 4 Discussion

The natural history of CHB infection is complicated, and patients can experience several stages of infection, changing from a state of high viral load without liver injury to an active state, then inactive, and then back to an active state after a few years. During the recurrent course of the disease, patients are at risk of serious complications such as liver fibrosis, cirrhosis, liver cancer, and even acute-on-chronic liver failure. Therefore, CHB is a dynamic condition with three main phases: 1) the immune tolerance phase associated with high HBV DNA, normal ALT levels, and no liver damage; 2) the immune active phase characterized by high HBV DNA, elevated ALT levels, and active liver inflammation; and 3) the inactive phase characterized by HBV DNA levels < 2000 IU/mL, normal ALT levels, and no liver damage. Patients infected with HBV can gradually move from one stage to the next with the possibility of reverting to a prior stage (35). As a result, this study recruited CHB patients without antiviral treatment and assessed the immune status of NK and CD8^+^ T cells from patients in different disease stages to guide stage-specific treatment approaches.

Distinct changes in the immune system occur during different phases of CHB infection, which helps to explain why studies showed conflicting findings on NK and CD8^+^ T cell function during this infection. In this study, we found that NK and CD8^+^ T cell exhaustion was more severe in active CHB patients than healthy subjects, and that NK cells with high Gal-9 expression could induce CD8^+^ T cell dysfunction through the Gal-9/TIM-3 pathway.

As an important component of the innate immune system, NK cells play a key role in the antiviral response (5). In chronic HBV infection, the persistence of the virus alters the NK cell phenotype and the expression of activating and inhibitory receptors on the NK cell surface. NKG2A is highly expressed in NK cells in patients with CHB, HCV, and hepatocellular carcinoma, resulting in NK cell dysfunction. Some studies showed that blocking NKG2A or antiviral therapy can reduce NKG2A expression and partially restore NK cell function (7, 36–39). In the current study, the proportion of NKG2A^+^ NK cells was elevated in the peripheral blood of CHB patients and significantly elevated in patients with active CHB, which is consistent with our previous findings (8). Recently, the expression of Gal-9 in NK cells has also been reported, and a high frequency of Gal-9^+^ NK cells was shown to reduce the effector capacity of NK cells (28, 29, 40). Gal-9 is widely expressed in a variety of human and mouse immune cells and exists in various sites including the cell membrane, cytoplasm, and nucleus (26). This study focused on the expression of Gal-9 on the surface of NK cells in CHB patients and found that there was significantly more Gal-9^+^ NK cells in CHB patients than in healthy controls, and the phenotype and function of Gal-9^+^ NK cells were markedly impaired relative to Gal-9^-^ NK cells. Moreover, the percentage of Gal-9^+^NK cells was lower in CHB patients who had received antiviral treatment for at least 6 months than in untreated patients. NKG2A expression was also reduced after treatment, which has been reported previously (7).

It has been demonstrated that the function of NK cells is impaired in chronic HBV infection, as shown by the reduced production of IFN-γ and TNF-α (7, 8). We further investigated and found that the expression of functional molecules, including IFN-γ, TNF-α, CD107a, granzyme B, and perforin, was also downregulated in circulating NK cells from CHB patients, primarily among those in the active phase.

As an important component of the adaptive immune system, CD8^+^ T cells also play a key antiviral role in HBV infection. However, unlike CD8^+^ T cells from patients with acute HBV infection, those from CHB patients gradually become weak or even lose their antiviral capacity as the virus persists. The virus-specific CD8^+^ T cell response can be heterogeneous depending on the disease phase (41, 42), with multilevel exhaustion. We found that circulating CD8^+^ T cells from CHB patients had reduced ability to produce effector cytokines, especially during the active phase, while those in other phases were still functional. Nebbia et al. (34) found that the response of HBV-specific CD8^+^ T cells was influenced by the level of HBV viral replication; therefore, the function of CD8^+^ T cells in CHB patients who had been receiving antiviral therapy for more than 6 months and were in a more stable state was assessed. We found that the function of CD8^+^ T cell was indeed significantly enhanced in these patients compared with untreated active CHB patients. The mechanism of CD8^+^ T cell exhaustion is complex, and the reduced effector function of exhausted CD8^+^ T cells is usually associated with elevated expression of inhibitory receptors, such as PD-1 (43) and TIM-3 (34). Blocking these inhibitory receptors *in vitro* can partially restore the function of HBV-specific CD8^+^ T cells. PD-1 and TIM-3 expression on CD8^+^ T cells from CHB patients were measured, and the proportion of TIM-3^+^CD8^+^ T cells was significantly increased. The proportion of PD-1^+^CD8^+^ T cells was also elevated, but not significantly (data not shown).

Several studies have shown that NK cells have both traditional antiviral and immunomodulatory effects and can regulate T cell immunity in multiple ways, including through NK cell receptors (18, 19), perforin (17), and cytokines (20–22), such as IFN-γ, IL-10. Iona S. Schuster et al. (44) found that TRAIL^+^ NK cells control CD4^+^ T cell responses during chronic viral infection to limit autoimmunity. However, few studies have assessed the regulation of CD8^+^ T cell immunity by NK cells in CHB patients. Peppa et al. (23) found that NK cells in CHB patients can induce virus-specific CD8^+^ T cell death through TNF-related apoptosis-inducing ligand (TRAIL) and negatively regulate antiviral immunity. In this study, we found that NK cells from CHB patients overexpress NKG2A and Gal-9. To determine whether CD8^+^ T cell immunity was regulated through these two molecules, further studies were conducted to demonstrate that CD8^+^ T cells from CHB patients overexpressed TIM-3, the Gal-9 natural receptor, while there was no difference in expression of HLA-E, the ligand for NKG2A. Moreover, the function of HBV-specific CD8^+^ T cells was restored after depletion of NK cells *in vitro*. Thus, it is likely that NK cells regulate CD8^+^ T cell immunity through the Gal-9/TIM-3 pathway.

The Gal-9/TIM-3 pathway has been reported to induce CD8^+^ T cell exhaustion in chronic viral infections, and blocking this pathway *in vitro* rescue CD8^+^ T cell exhaustion. Moreover, the Gal-9/TIM-3 pathway was reported as a promising target for immunotherapy (31, 33, 45). We cocultured Gal-9^+^ NK and CD8^+^ T cells *in vitro* and demonstrated that Gal-9^+^ NK cells can promote CD8^+^ T cell exhaustion in CHB patients. Furthermore, stimulating PBMCs with HBV peptide and blocking the Gal-9/TIM-3 pathway partially restored HBV-specific CD8^+^ T cell function. In addition, we purified NK cells and CD8^+^ T cells from active CHB patients and cocultured them *in vitro*, and blocking Gal-9/TIM-3 partially rescued CD8^+^ T cells exhaustion.

In summary, our study demonstrates a mechanism by which NK cells induce CD8^+^ T cells dysfunction. The findings indicate that Gal-9/TIM-3 axis is a potential therapeutic checkpoint in CHB patients.

## Data Availability Statement

The raw data supporting the conclusions of this article will be made available by the authors, without undue reservation.

## Ethics Statement

The studies involving human participants were reviewed and approved by the local ethics committee of The First Affiliated Hospital of Anhui Medical University. The patients/participants provided their written informed consent to participate in this study.

## Author Contributions

SL and CX performed the experiments, analysed the data and wrote the manuscript. FY and YQ were involved in data analysis. TL and YL were involved in the collection of clinical samples. YX and LZ critically reviewed the manuscript. YG and QS supplied and evaluated CHB patients. MZ designed and supervised the experiments and wrote the manuscript. All authors contributed to the article and approved the submitted version.

## Funding

This work was supported by National Natural Science Foundation of China (81771685) and Anhui Medical University Basic and Clinical Cooperative Research Promotion Program (2019xkjT023).

## Conflict of Interest

The authors declare that the research was conducted in the absence of any commercial or financial relationships that could be construed as a potential conflict of interest.

## Publisher’s Note

All claims expressed in this article are solely those of the authors and do not necessarily represent those of their affiliated organizations, or those of the publisher, the editors and the reviewers. Any product that may be evaluated in this article, or claim that may be made by its manufacturer, is not guaranteed or endorsed by the publisher.
