# Supplementary material for: Natural Killer Cells Induce CD8+ T Cell Dysfunction via Galectin-9/TIM-3 in Chronic Hepatitis B Virus Infection
Source: Front Immunol. 2022 Jun 28;13:884290. doi: 10.3389/fimmu.2022.884290 (PMC9301626; doi:10.3389/fimmu.2022.884290)
Supplement: Supplementary file 1 [file DataSheet_1.docx]

Supplementary Material

# Supplementary Figures


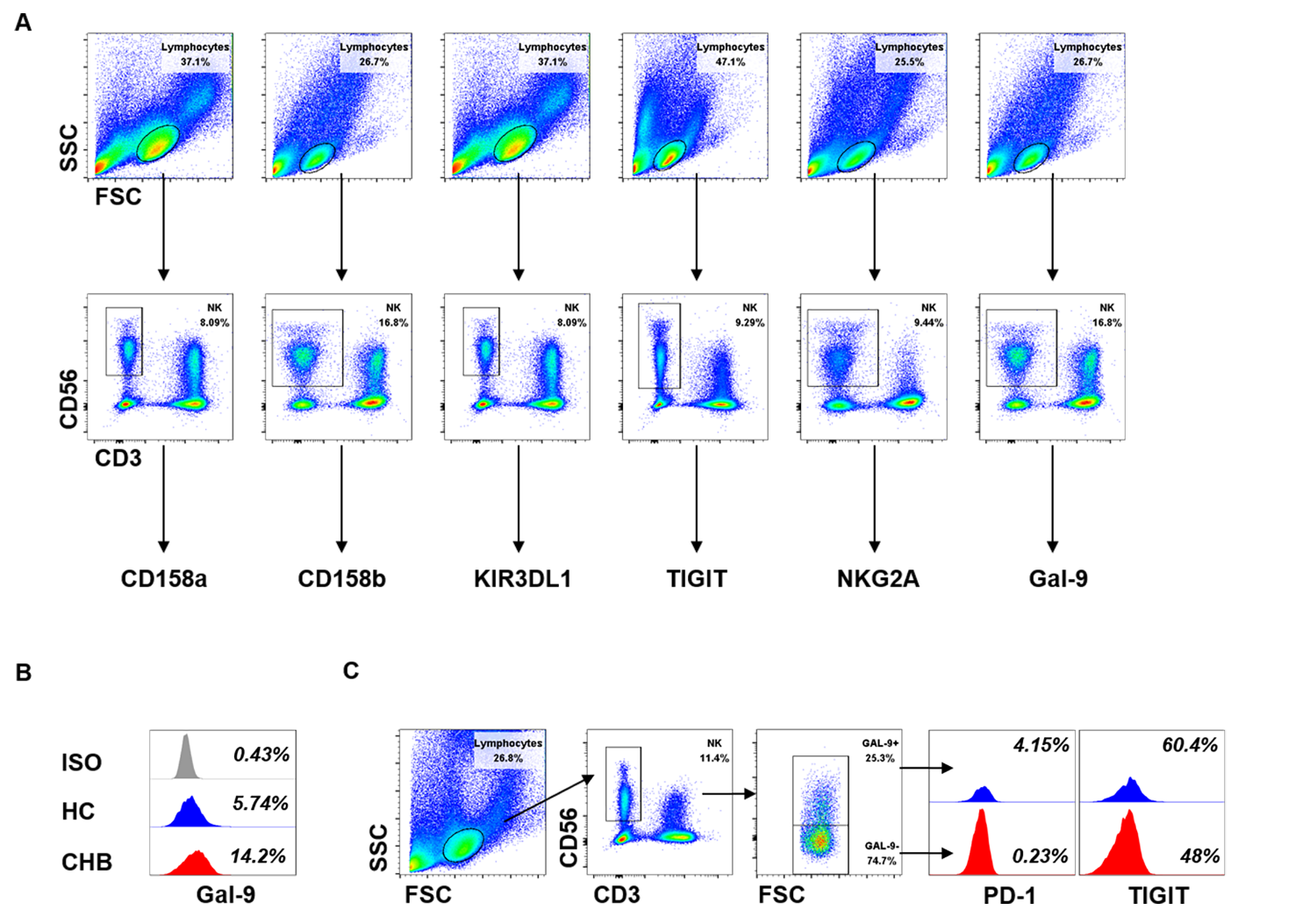


**Supplementary Figure 1. Gating information in Figure 1.** **(A)** Gating strategy in Figure 1A. **(B)** The Gal-9 expression gating strategy. **(C)** Gating strategy in Figure 1D, 1E.


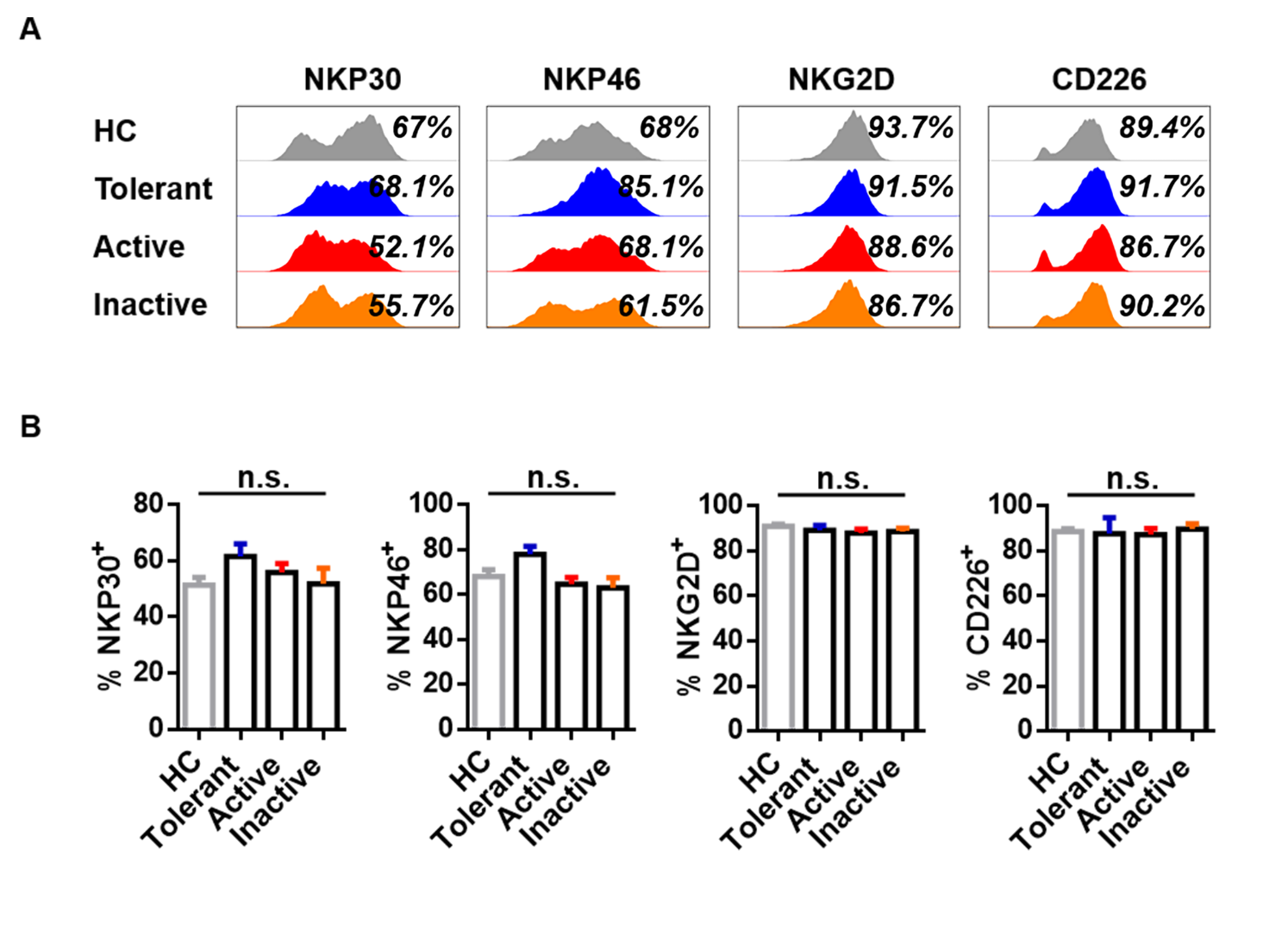


**Supplementary Figure 2. The expression of activating receptors on NK cells in CHB patients. (A)** Representative flow cytometry plots showing the expression of the activating receptors NKP30, NKP46, NKG2D, and CD226 on circulating NK cells in Tolerant, Active, and Inactive CHB patients and HCs. **(B)** Comparison of the percentages of receptors expressed on circulating NK cells in **(A)**. Results are expressed as mean ± SEM, and the number of samples (n) in each group was ≥ 3. Two-by-two comparisons between the four groups were performed by one-way ANOVA test. The groups with significant differences are marked, while the unmarked paired groups have no differences between them. n.s.: not significant.


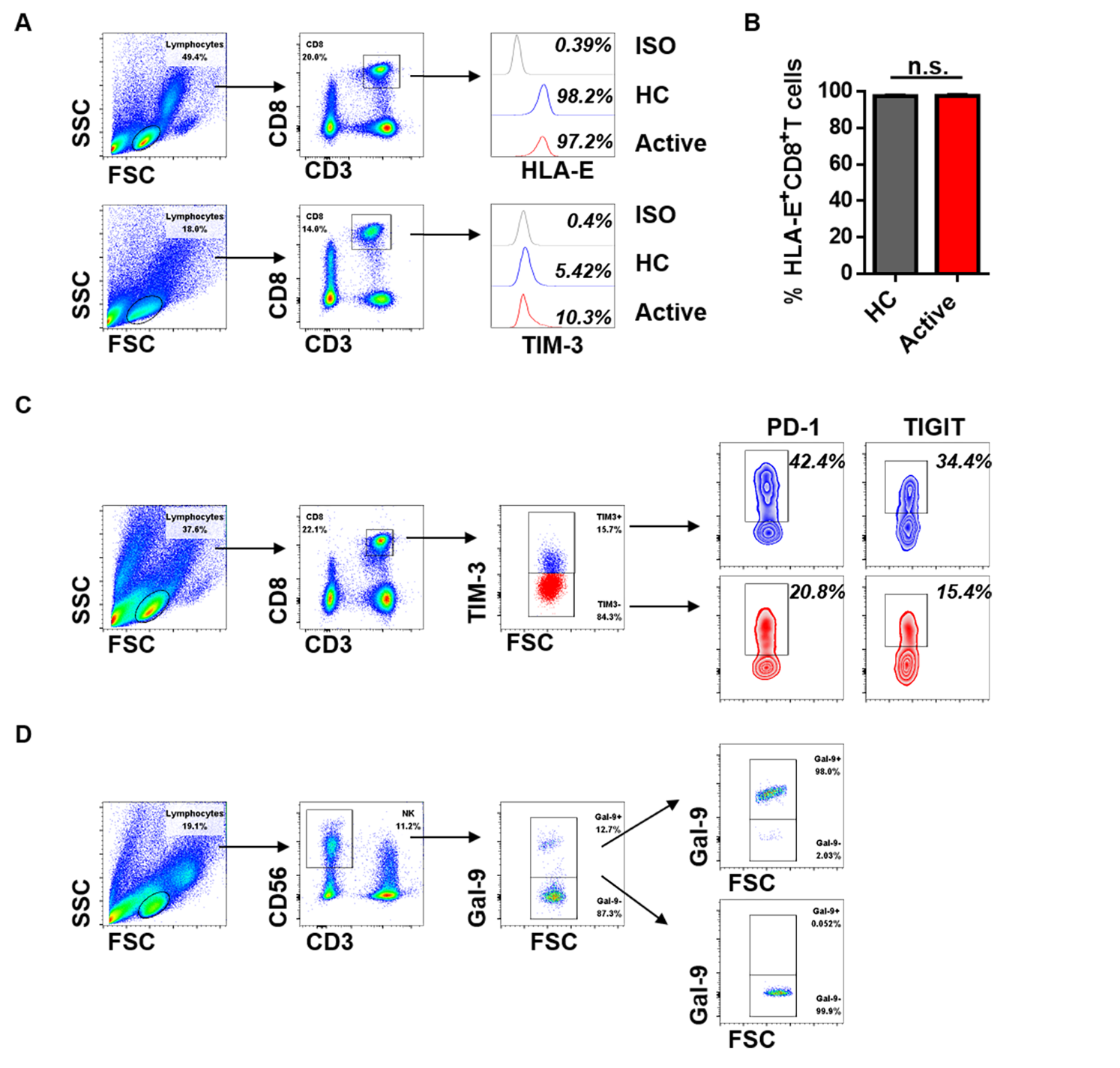


**Supplementary Figure 3. Gating information in Figure 4.** **(A)** The gating strategy to identify HLA-E and TIM-3 expression on CD8^+^ T cells in Figure 4A, Supplementary Figure 3B. **(B)** Comparison of the percentage of HLA-E expressed in circulating CD8^+^ T cells in active CHB patients and HCs. **(C)** The gating strategy of Figure 4B, 4C. **(D)** The gating strategy for Gal-9^+/-^ NK cells sorting and the representative flow cytometry plots showing the purity of Gal-9^+/-^ NK cells after sorting.


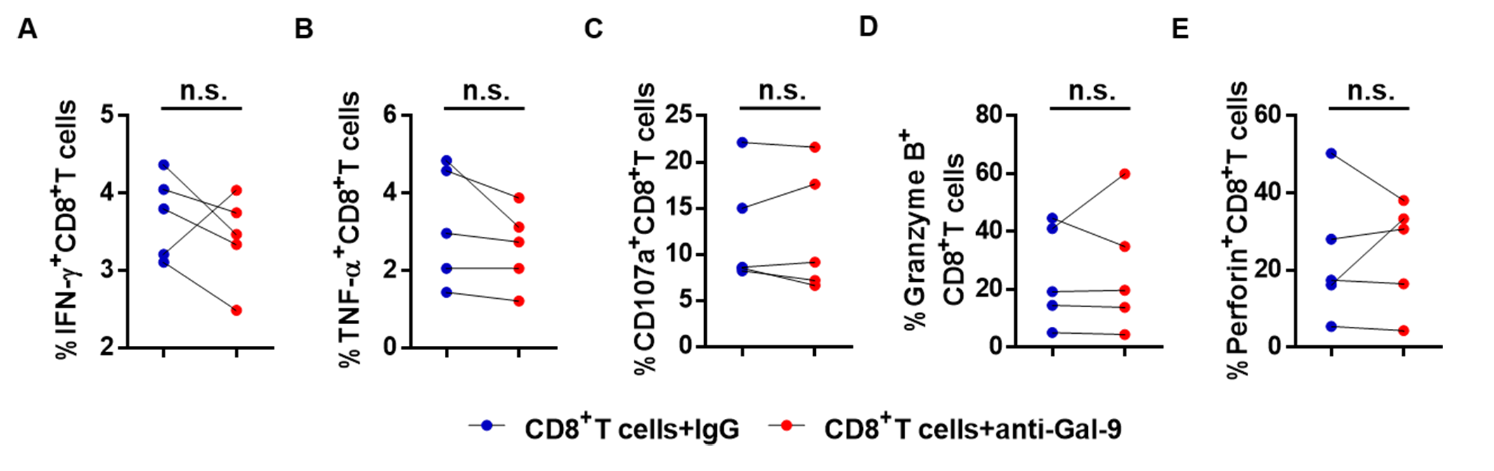


**Supplementary Figure 4. The effect of the Gal-9 blocking requires NK cells.** **(A-E)** CD8^+^ T cells from active CHB patients were sorted and treated with anti-Gal-9 or control IgG *in vitro*, stimulated with anti-CD3/anti-CD28 for 3 days. The production of the cytokines IFN-γ, TNF-α, CD107a, granzyme B, and perforin by CD8^+^ T cells was analyzed. Results are expressed as the mean ± SEM, and the number of samples (n) in each group was ≥ 3. Paired t-test was used to compare paired samples. n.s.: not significant.
